# Proteomic Profiling and In Silico Characterization of the Secretome of *Anisakis simplex* Sensu Stricto L3 Larvae

**DOI:** 10.3390/pathogens11020246

**Published:** 2022-02-14

**Authors:** Maciej Kochanowski, Joanna Dąbrowska, Mirosław Różycki, Jacek Sroka, Jacek Karamon, Aneta Bełcik, Weronika Korpysa-Dzirba, Tomasz Cencek

**Affiliations:** Department of Parasitology and Invasive Diseases, National Veterinary Research Institute, Partyzantów Avenue 57, 24-100 Puławy, Poland; joanna.dabrowska@piwet.pulawy.pl (J.D.); mrozycki@piwet.pulawy.pl (M.R.); jacek.sroka@piwet.pulawy.pl (J.S.); j.karamon@piwet.pulawy.pl (J.K.); aneta.belcik@piwet.pulawy.pl (A.B.); weronika.korpysa@piwet.pulawy.pl (W.K.-D.); tcencek@piwet.pulawy.pl (T.C.)

**Keywords:** allergen, bioinformatics, *Anisakis simplex*, pathogenicity, proteomics, secretome

## Abstract

*Anisakis simplex* sensu stricto (s.s.) L3 larvae are one of the major etiological factors of human anisakiasis, which is one of the most important foodborne parasitic diseases. Nevertheless, to date, *Anisakis* secretome proteins, with important functions in nematode pathogenicity and host-parasite interactions, have not been extensively explored. Therefore, the aim of this study was to identify and characterize the excretory-secretory (ES) proteins of *A. simplex* L3 larvae. ES proteins of *A. simplex* were subjected to liquid chromatography-tandem mass spectrometry (LC-MS/MS) analysis, and the identified proteins were then analyzed using bioinformatics tools. A total of 158 proteins were detected. Detailed bioinformatic characterization of ES proteins was performed, including Gene Ontology (GO) analysis, identification of enzymes, Kyoto Encyclopedia of Genes and Genomes (KEGG) pathways analysis, protein family classification, secretory pathway prediction, and detection of essential proteins. Furthermore, of all detected ES proteins, 1 was identified as an allergen, which was Ani s 4, and 18 were potential allergens, most of which were homologs of nematode and arthropod allergens. Nine potential pathogenicity-related proteins were predicted, which were predominantly homologs of chaperones. In addition, predicted host-parasite interactions between the *Anisakis* ES proteins and both human and fish proteins were identified. In conclusion, this study represents the first global analysis of *Anisakis* ES proteins. The findings provide a better understanding of survival and invasion strategies of *A. simplex* L3 larvae.

## 1. Introduction

*Anisakis simplex* sensu stricto (s.s.), a nematode species belonging to the family Anisakidae, is among the most important foodborne parasites capable of causing a disease in humans called anisakiasis. This roundworm has an indirect lifecycle involving several hosts. Marine mammals are definitive hosts, fish, and cephalopods are intermediate or paratenic hosts, while crustaceans are intermediate hosts [1,2]. Humans, which are accidental hosts of *A. simplex*, are infected by third-stage (L3) larvae of this nematode, the source of which are infected marine fish or cephalopods [1,3]. *A. simplex* (s.s.) is mainly distributed in the northern Atlantic and Pacific Oceans [1]. However, other areas of occurrence (e.g., western Mediterranean Sea) were also reported [1,4,5].

The global incidence of anisakiasis is 0.32 cases/100,000 inhabitants [6]. However, according to recent studies, the prevalence of the disease is estimated to be much higher [7]; this discrepancy is linked to the nonspecificity of symptoms and the limitations of diagnostic tools. Furthermore, anisakiasis has become increasingly more important as human health risk, especially in regions where the consumption of raw or only lightly processed fish and seafood is frequent [1,8,9]. Therefore, the majority of anisakiasis cases are noted in Southeast Asia, while cases in Europe are less common and occur mainly in Spain and Italy [1].

Anisakiasis is a parasitic disease that affects the gastrointestinal tract [1,4]. In such cases, patients typically have symptoms such as abdominal pain, nausea, and vomiting [10]. Occasionally, the larva may perforate the alimentary tract and migrate into the peritoneal cavity and internal organs [1]. Another very important form of the disease is gastroallergic anisakiasis, in which abdominal symptoms are accompanied by allergic-like reactions such as urticaria, angioedema, or even anaphylaxis [1,11]. Furthermore, studies have suggested that dead *Anisakis* larvae can cause allergic reactions in sensitized humans [12,13,14]. From an anatomopathological point of view, *Anisakis* larva invasion leads to the development of inflammation (predominantly eosinophilic), edema, hemorrhage, formation of granuloma, and ulcers at the larval location, which is usually in the mucosa and submucosa of the gastrointestinal wall [10,12]. Larvae are unable to develop in the human body and die after up to 3 weeks [15].

Because of the risk to human health described above, *Anisakis* L3 larvae have been the subject of many studies, including investigations of their pathogenicity [16,17,18] and host-parasite interactions [19,20,21]. Excretory-secretory (ES) proteins play a key role in these activities. These molecules react with host proteins and enable development of host-parasite relationships by regulating the host immune system and subsequent pathology associated with the immune response. Furthermore, ES proteins are crucial for the parasite survival in host by inhibiting the inflammatory reactions [22]. Secretome proteins are important components of the structure and metabolism of the parasite, and exploration of *A. simplex* secretome will identify candidate molecules involved in immune modulation. Furthermore, these proteins are often very valuable targets for diagnostic tests, drugs, or vaccines. Nevertheless, the secretome proteins of *A. simplex* and other nematodes belonging to the family Anisakidae have not been extensively explored to date, which has resulted in a massive knowledge gap. 

One of the most powerful tools used for proteomic profiling is mass spectrometry. This technology, combined with the use of an appropriate protein database, is suitable for the sensitive identification in complex biological samples. The potential for proteomic identification by mass spectrometry was recently greatly enhanced by the improved availability of genome sequencing technology, which has resulted in increased protein sequence database resources. Therefore, mass spectrometry has been successfully used for the proteomic profiling of secretomes of many organisms, including several nematode species, such as *Toxocara canis* [23], *Ascaris suum* [24], *Ancylostoma caninum* [25], and *Spirocerca lupi* [26].

In light of the above considerations, the aim of this study was to conduct proteomic profiling of the *A. simplex* (s.s.) L3 secretome. The use of liquid chromatography-tandem mass spectrometry (LC-MS/MS) allowed for the high-throughput identification of ES proteins. Multiple bioinformatics tools were used to comprehensively characterize the detected *Anisakis* ES proteins. Furthermore, in the present study, particular focus was placed on the discovery of proteins involved in pathogenesis of anisakiasis and host-pathogen interactions.

## 2. Results

### 2.1. Mass Spectrometry-Based Identification 

LC-MS/MS analysis resulted in the identification of 158 proteins that occurred in all biological replicates. All protein identifications were based on the presence of at least one unique peptide. Of all identified proteins, 85 had at least two unique peptides detected in three biological replicates. The molecular weights (MWs) of proteins ranged from 4 to 811 kDa, and their isoelectric points (pI) ranged from 4 to 8.99. The majority of the proteins had MWs below 50 kDa (116 proteins) and a pI below 6.6 (107 proteins). All of the 158 proteins analyzed in this study are listed in Supplementary File S1.1. Mascot results of proteins identification within individual biological replicates are presented in Supplementary File S1.2.

### 2.2. Comparison of A. simplex Secretome Proteins with Secretome Proteins of Other Nematodes

**Figure 1:**
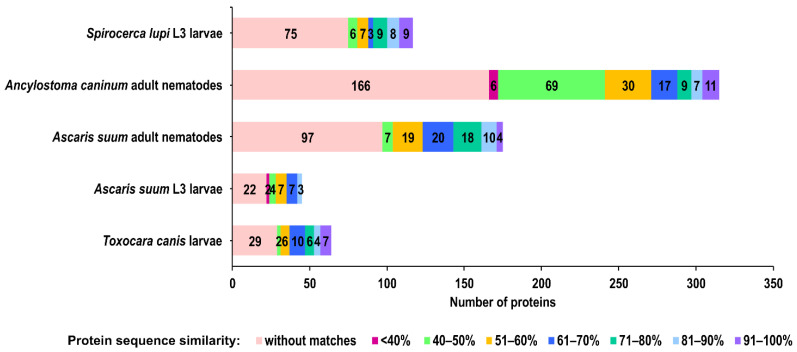
BLASTP-based comparisons of the similarity of the secretome proteins of selected nematodes to secretome proteins of *A. simplex* (s.s.) L3 larvae.

The secretome proteins of *A. simplex* (s.s.) were compared with those of selected nematodes to determine similarities and differences in their profiles. The following secretomes had the largest number of highly similar proteins (≥70% similarity) to those of the *A. simplex* secretome: adult *Ascaris suum* (32 proteins), adult *Ancylostoma caninum* (27 proteins), and L3 larvae of *Spirocerca lupi* (26 proteins). Fewer highly similar proteins were detected in the secretomes of *Toxocara canis* larvae (17 proteins) and *Ascaris suum* L3 larvae (three proteins). Among the highly similar proteins, homologs of the following *A. simplex* proteins were the most common: peptidyl-prolyl cis-trans isomerase (A0A0M3JT42), which has homologs in adult *A. caninum*, adult *A. suum*, L3 larvae of *A. suum*, and L3 larvae of *S. lupi*, and an uncharacterized protein (A0A0M3JWS2) and putative actin (A0A0M3J0M4), both of which have homologs in adult *A. caninum*, adult *A. suum*, L3 larvae of *S. lupi*, and *T. canis* larvae. Details of the comparison of secretome proteomic profiles are presented in Figure 1 and Supplementary Files S1.3–8.

### 2.3. Protein Family Classification

The proteins in the *A. simplex* (s.s.) secretome belong to different families, among which the following were most frequently represented: immunoglobulin-like fold (15 proteins), immunoglobulin-like domain superfamily (12 proteins), annexin superfamily (six proteins), and thioredoxin-like superfamily (five proteins). Most protein families (87 protein families) were represented by only one protein. A total of 143 protein families were identified (see Supplementary File S1.9).

### 2.4. Secretory Pathway Prediction

Based on bioinformatics prediction, 21 proteins with identified signal peptides were classified into the conventional secretory pathway, and 77 proteins were assigned to unconventional protein secretion. Furthermore, among ES proteins the following proteins known to be associated with extracellular vesicles (EVs) released from *Anisakis* [27] were found: putative actin (A0A0M3J0M4), heat shock 70 kDa protein cognate 1 (A0A0M3K9V2), glutamate dehydrogenase (NAD(P)(+)) (A0A0M3K4H2), uncharacterized protein (A0A0M3KAB8), superoxide dismutase [Cu-Zn] (A0A0M3J718), and pepsin-I3 domain-containing protein (A0A0M3JAH0). Additionally, 24 proteins probably EV-associated were found in the *Anisakis* secretome. These protein are homologs of EV-associated protein which were identified in the secretomes of the following nematodes: *A. suum* [28], *Brugia malayi* [29], and *Nippostrongylus brasiliensis* [30]. Among the potential EV-associated proteins, the best matches were as follows: proteasome subunit alpha type (A0A0M3K144), proteasome subunit alpha type-3 (A0A0M3JSH7), and triosephosphate isomerase (A0A0M3JVA5). The top 10 best matches from identification of proteins potentially associated with EV are shown in Table 1, and all the results from the secretory pathway and EV-associated protein predictions are presented in Supplementary Files S1.10–12.

### 2.5. Gene Ontology (GO) Annotation and Enrichment Analysis

**Figure 2:**
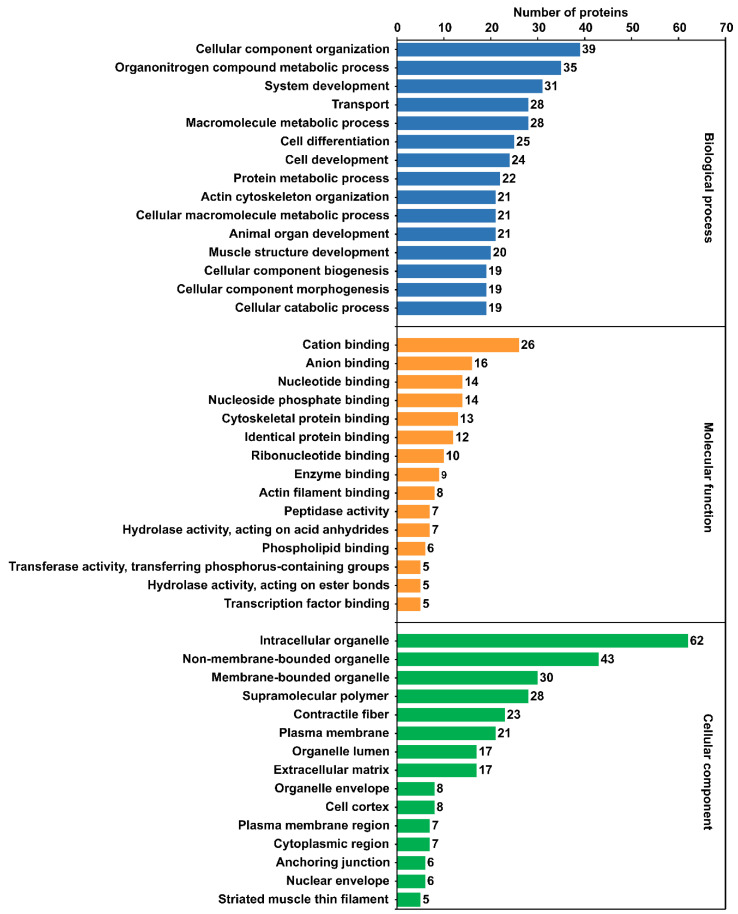
Distribution of the top 15 most abundant Gene Ontology (GO) terms in the categories of biological process, molecular function, and cellular component.

GO annotations of identified *A. simplex* (s.s.) proteins were grouped into three categories: biological process, molecular function, and cellular component. Around 90% of the proteins were annotated with GO terms. A total of 1242 GO annotations were identified. Only 10 proteins were assigned a single GO annotation, and the remaining proteins were annotated with 2-60 GO terms. In the biological process category, the most frequent GO terms were cellular component organization (39 proteins), organonitrogen compound metabolic process (35 proteins), and system development (31 proteins). In the molecular function category, the following GO terms were the most abundant: cation binding (26 proteins), anion binding (16 proteins), nucleotide binding (14 proteins), and nucleoside phosphate binding (14 proteins). The most abundant terms in the cellular component category were as follows: intracellular organelle (62 proteins), nonmembrane-bounded organelle (43 proteins), and membrane-bounded organelle (30 proteins). The top 15 GO terms in the three ontology categories are shown in Figure 2, and all GO annotations for individual proteins are listed in Supplementary File S1.13.

**Figure 3:**
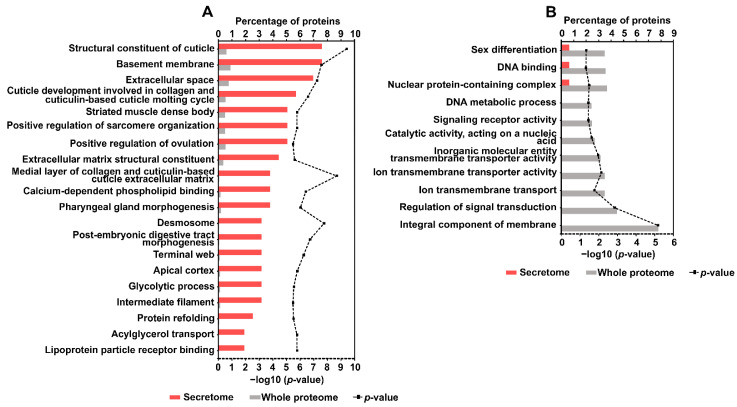
The top 20 overrepresented (**A**) and all underrepresented (**B**) GO terms in descending order of frequency in the secretome; bars show the percentages of proteins in the secretome and the whole proteome of *A. simplex* that are associated with the GO term; calculated GO enrichment *p*-values (−log10(*p*-value)) are plotted in the chart.

Enrichment analysis allowed for the mapping of over- and underrepresented GO terms of *A. simplex* (s.s.) secretome proteins. This analysis was performed by comparing the representation of GO terms for the detected secretome proteins with that for the whole *A. simplex* proteome. A total of 174 overrepresented and 11 underrepresented GO terms were identified. According to the calculated *p*-values, the following GO terms were the most overrepresented: structural constituent of cuticle (*p*-value = 3.88 × 10^−10^), medial layer of collagen and cuticulin-based cuticle extracellular matrix (*p*-value = 2.02 × 10^−9^), and desmosome (*p*-value = 1.81 × 10^−8^). Conversely, the following GO annotations were the most underrepresented: integral component of membrane (*p*-value = 6.57 × 10^−6^), regulation of signal transduction (*p*-value = 0.001), and ion transmembrane transporter activity (*p*-value = 0.007). The top 20 overrepresented and underrepresented GO terms are shown in Figure 3A,B, respectively. The detailed results of the GO enrichment analysis are shown in Supplementary File S1.14.

### 2.6. Enzyme Identification and Enrichment Analysis

**Figure 4:**
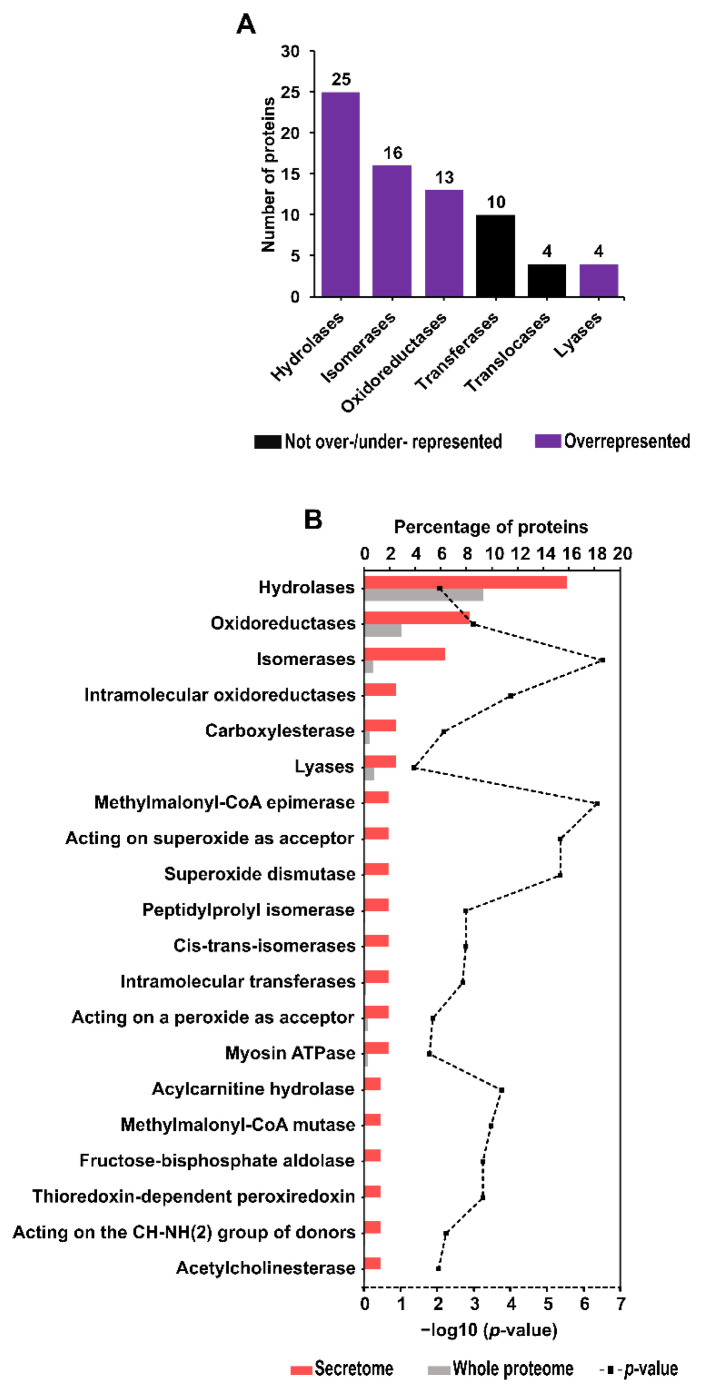
Enzyme class identification results (**A**). The top 20 overrepresented enzymes in descending order of frequency in the secretome; bars show the percentages of proteins in the secretome and the whole proteome of *A. simplex* that are associated with the enzymes; calculated enzyme enrichment *p*-values (−log10(*p*-value)) are plotted in the chart (**B**).

Bioinformatics analysis of *A. simplex* (s.s.) secretome proteins was used to identify enzymes and proteins involved in metabolic pathways. Seventy-two proteins were assigned to six enzyme classes. The most abundant class of enzymes was hydrolases (25 proteins), and the less represented classes were isomerases, transferases, oxidoreductases, translocases, and lyases. No proteins belonging to the ligase class were detected. The distribution of the number of proteins in each enzyme class is shown in Figure 4A.

As determined using the OmicsBox software, 38 enzymes (including enzyme classes/subclasses) were overrepresented in the secretome compared to the whole *A. simplex* proteome. Based on the calculated P-values, the most overrepresented enzymes were isomerases (*p*-value = 3.08 × 10^−7^), methylmalonyl-CoA epimerase (*p*-value = 4.31 × 10^−7^), acting on superoxide as acceptor (*p*-value = 4.26 × 10^−6^), and superoxide dismutase (*p*-value = 4.26 × 10^−6^). No underrepresented enzymes were found in the *Anisakis* secretome. Figure 4B shows the 20 overrepresented enzymes in descending order of their abundance in the secretome. Details of the enzyme enrichment analysis are presented in Supplementary File S1.15.

In addition, proteases and protease inhibitors were identified using the MEROPS database. According to the used BLAST search cutoff, 36 of these enzymes were found, of which 27 were proteases and nine were protease inhibitors. Eighteen proteases/protease inhibitors showed 100% similarity to *A. simplex* proteins reported in the MEROPS database. A large group (eight proteins) also includes proteins that show similarity to proteases/protease inhibitors of other parasitic helminths, such as *A. suum*, *Trichuris suis*, *Trichinella nativa*, *Onchocerca volvulus*, *Hymenolepis nana*, and *Hymenolepis diminuta*. Table 2 shows the top 10 secretome protein matches of proteases/protease inhibitors, and all results are presented in Supplementary File S1.16.

### 2.7. Kyoto Encyclopedia of Genes and Genomes (KEGG) Pathway Identification and Enrichment Analysis

**Figure 5:**
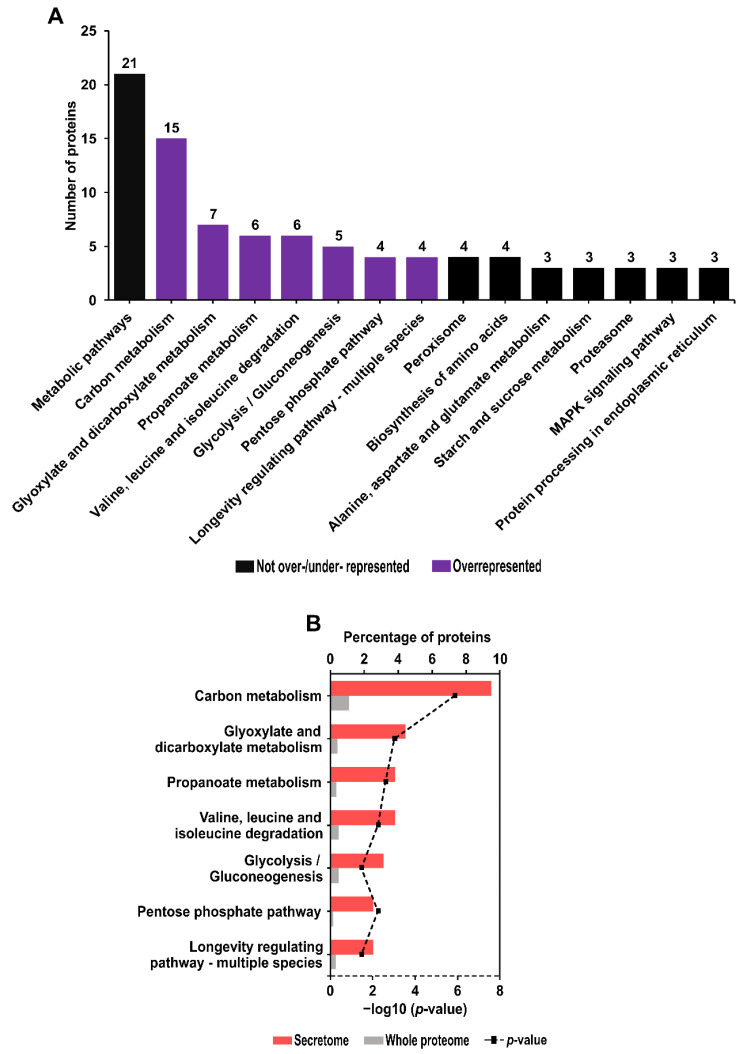
The top 15 most abundant KEGG pathways (**A**). Overrepresented KEGG pathways in descending order of frequency in the secretome; bars show the percentages of proteins in the secretome and the whole proteome of *A. simplex* that are associated with the enzymes are shown; calculated enzyme enrichment *p*-values (−log10(*p*-value)) are plotted in the chart (**B**).

The KEGG pathway profiling of *Anisakis* secretome proteins revealed proteins involved in 44 pathways. Among them, the most frequent were metabolic pathways (21 proteins), followed by carbon metabolism (15 proteins), and glyoxylate and dicarboxylate metabolism (seven proteins). The 15 most abundant KEGG pathways are shown in Figure 5A. The other 29 KEGG pathways were represented by 1–2 proteins. All identified KEGG pathways are listed in Supplementary File S1.17.

KEGG pathway enrichment analysis revealed seven overrepresented pathways in the secretome compared to the whole *A. simplex* proteome (Figure 5B). In ascending order of the calculated *p*-value, the most overrepresented KEGG pathways were as follows: carbon metabolism (*p*-value = 1.39 × 10^−6^), glyoxylate and dicarboxylate metabolism (*p*-value = 0.00097), and propanoate metabolism (*p*-value = 0.00253). No underrepresented KEGG pathways were found. Detailed results of the KEGG pathway enrichment analysis are presented in Supplementary File S1.17.

### 2.8. Identification of Essential Proteins 

Among the identified secretome proteins of *A. simplex* (s.s.), 33 essential proteins were predicted (see Supplementary File S1.18) using the DEG database. Essential proteins are those indispensable for the survival of an organism. These proteins belong to various protein families. The best three matches against the sequences from the database were as follows: putative actin (A0A0M3J0M4), calmodulin (A0A0M3K916), and an uncharacterized protein (A0A0M3K916) that shows homology to alpha-actinin-4. Table 3 presents the 10 *Anisakis* proteins with the highest similarity to known essential proteins.

### 2.9. Identification of Potential Pathogenicity-Related Proteins 

**Figure 6:**
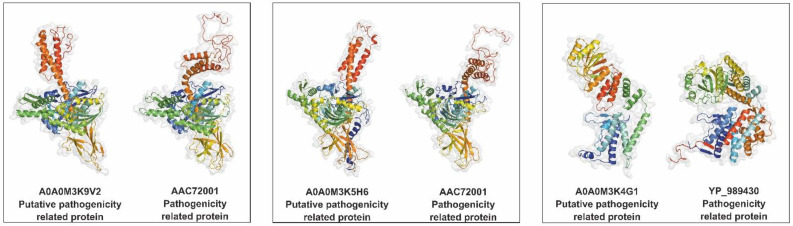
Comparison of the predicted tertiary structures of the 3 top-ranked putative pathogenicity-related proteins against their homologs with confirmed pathogenic properties. UniProt accession numbers of putative pathogenicity-related proteins and GenBank accession numbers of pathogenicity-related proteins are displayed in the figure.

Using three databases, nine putative pathogenicity-related proteins were identified in the *A. simplex* (s.s.) secretome. Four proteins were found in multiple databases, and five proteins were detected in a single database. The highest number of potential pathogenicity-related proteins (eight proteins) was identified using the VICTORS database. Hits with the highest similarity to confirmed pathogenicity-related proteins were as follows: heat shock 70 kDa protein cognate 1 (A0A0M3K9V2), 78 kDa glucose-regulated protein (A0A0M3K5H6), and an uncharacterized protein (A0A0M3K4G1). The 3D structures reveal similarities between these proteins and their homologs with confirmed pathogenic properties (see Figure 6). A relatively high number of detected potential pathogenicity-related proteins (A0A0M3K9V2, A0A0M3K5H6, A0A0M3K4G1, and A0A0M3K0Q9) have homologs in the heat shock protein (HSP) family. Furthermore, the majority of the putative pathogenicity-related proteins in the *Anisakis* secretome (five proteins) are homologs of bacterial virulence proteins. Three *A. simplex* proteins show similarity to *Cryptococcus neoformans* virulence proteins, and two are homologs of *Toxoplasma gondii* HSP. All of the pathogenicity-related proteins identified in the study are shown in Table 4.

### 2.10. Allergen and Potential Allergen Identification

**Figure 7:**
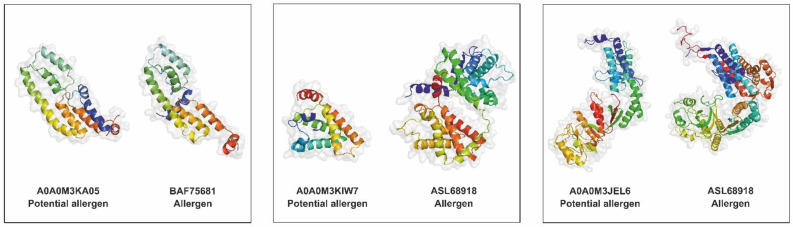
Comparison of the predicted tertiary structures of the 3 top-ranked potential allergens against their homologs with confirmed allergen properties. UniProt accession numbers of potential allergens and GenBank accession numbers of allergens are displayed in the figure.

Of all identified proteins, only one (Ani s 4) is listed by the World Health Organization and the International Union of Immunological Societies (WHO/IUIS) Allergen Nomenclature Sub-Committee. By contrast, using the FARRP database, 18 potential allergens were identified. The three proteins with the best identification against the FARRP database were as follows: SXP/RAL-2 family protein 2 isoform 1 (A0A0M3KA05) and two globin-like proteins (A0A0M3KIW7, A0A0M3JEL6). The 3D structures of these potential allergens in comparison with their homologous allergens are shown in Figure 7. The AllerCatPro server confirmed that all proteins identified using the FARRP database have possible allergenic potential. The AllerCatPro tool determined the allergenic properties of 11 proteins with high confidence and 7 proteins with low confidence. The five detected potential allergens showed high similarity (>92%) to *A. simplex* allergens, and four others are highly similar to mite allergens. The other detected putative allergens showed similarity to allergens of *A. suum*, mosquito, fish, freshwater crayfish, and fungus. All potential allergens found in the *A. simplex* (s.s.) secretome are presented in Table 5.

### 2.11. Predicted Protein-Protein Interactions in A. simplex (s.s.) Secretome

**Figure 8:**
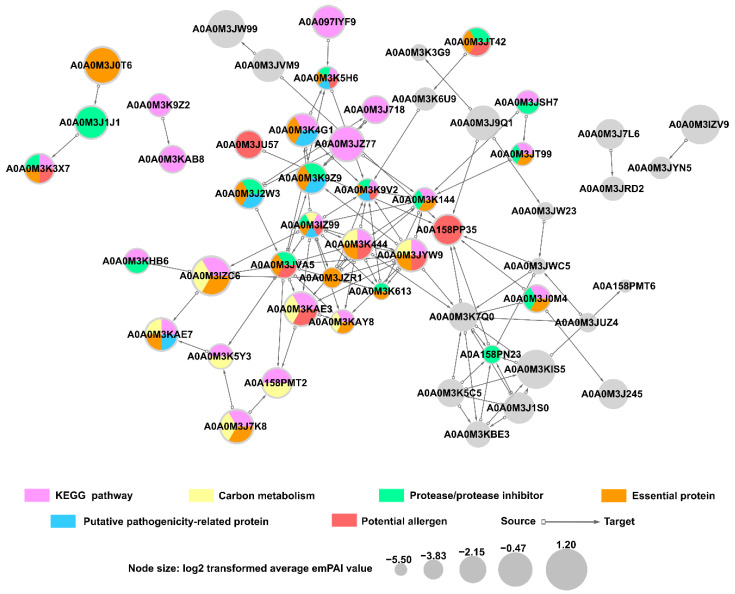
Analysis of the protein-protein interaction network in *A. simplex* (s.s.) secretome. UniProt accession numbers of proteins are displayed in the interaction network.

The protein interaction network was established using STRING and showed predicted interactions between ES proteins of *A. simplex* (s.s.). Fifty-three proteins involved in the interaction network were revealed with high prediction confidence. Twenty-five of these proteins were associated with KEGG metabolic pathways, particularly carbon metabolism, in which ten proteins were involved. Furthermore, proteins involved in the interaction network were associated with the following groups: essential proteins (21 proteins), proteases/protease inhibitors (16 proteins), potential allergens (11 proteins), and potential pathogenicity-related proteins (seven proteins). Twenty-nine proteins were assigned to only one of the groups listed above, and eleven were categorized into several groups simultaneously. Nineteen proteins from the interaction network were not assigned to any of the explored groups. The detailed analysis of the protein interaction network is shown in Figure 8.

### 2.12. Predicted Host-Parasite Protein Interactions

The HPIDB 3.0 server was used for the prediction of host-parasite interactions between the *A. simplex* (s.s.) ES proteins and both human and fish (Atlantic herring) proteins.

**Figure 9:**
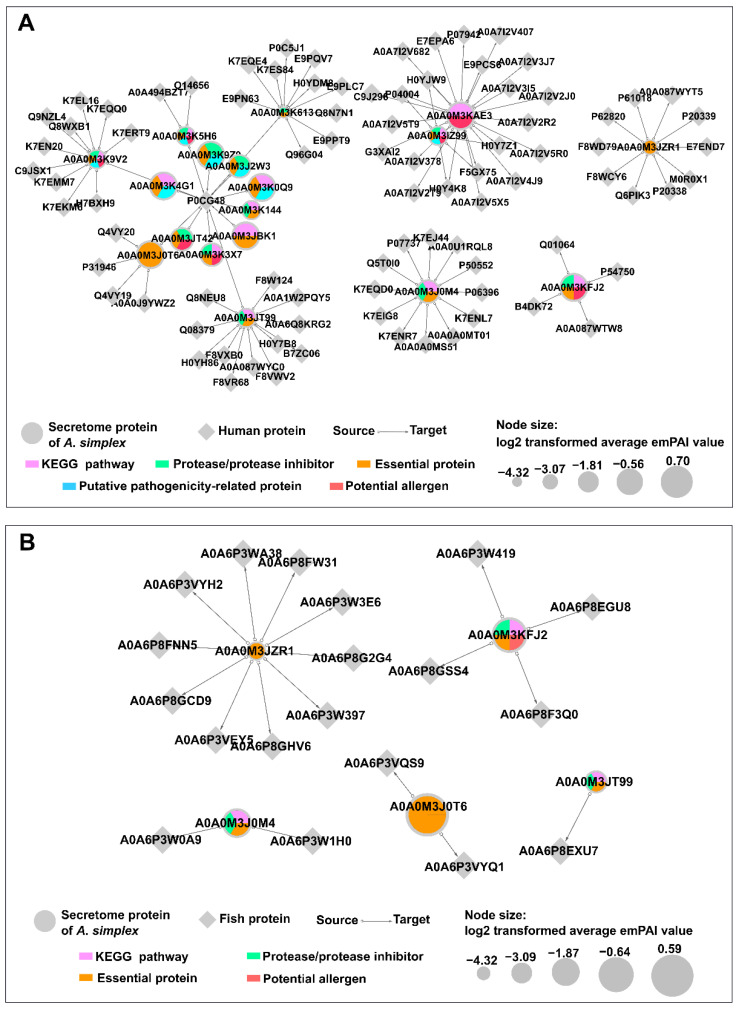
Analysis of the predicted protein-protein interactions between the *A. simplex* (s.s.) secretome and hosts. Interactions with human proteins are presented in part (**A**), and interactions with fish (Atlantic herring) proteins are presented in part (**B**). UniProt accession numbers of proteins are displayed in the interaction network.

Eighteen proteins of *Anisakis* and 87 human proteins were identified in the host-parasite interaction network (see Figure 9A). The following groups of proteins were detected among *Anisakis* secretome proteins involved in interactions with human proteins: essential proteins (17 proteins), proteases/protease inhibitors (12 proteins), KEGG pathway proteins (12 proteins), potential allergens (seven proteins), and potential pathogenicity-related proteins (seven proteins). Most secretome proteins (16 proteins) were classified into two or more of these groups. Furthermore, it should be noted that the following *Anisakis* ES proteins showed the highest number of potential interactions with human proteins: transaldolase (A0A0M3KAE3; 22 interactions), proteasome subunit alpha type (A0A0M3JT99; 13 interactions), putative actin (A0A0M3J0M4; 12 interactions), heat shock 70 kDa protein cognate 1 (A0A0M3K9V2; 11 interactions), elongation factor 2 (A0A0M3K613; 11 interactions), and Rab GDP dissociation inhibitor (A0A0M3JZR1; 10 interactions). Human proteins that were predicted to be involved in the host-parasite interaction network belong to many different families, and the most highly represented of them were the following: laminins (17 proteins), methyltransferase proteins (13 proteins), Ras-related proteins (nine proteins), and Hsp70-binding proteins (seven proteins). Furthermore, among human proteins, polyubiquitin-C (P0CG48) showed potential interactions with the highest number of *Anisakis* proteins (13 interactions).

Five proteins of *Anisakis* secretome and 19 proteins of Atlantic herring were predicted in the fish-parasite interaction network (see Figure 9B). All *Anisakis* ES proteins identified in this interactome were identified also in the human-parasite interaction network. The following groups of proteins were detected among *Anisakis* ES proteins involved in interactions with fish proteins: essential proteins (five proteins), proteases/protease inhibitors (3 proteins), KEGG pathway proteins (three proteins), and potential allergen (one protein). Rab GDP dissociation inhibitor (A0A0M3JZR1), and calmodulin (A0A0M3KFJ2) showed potential interactions with the highest number of fish proteins (10 and four interactions, respectively). The most highly represented fish proteins involved in the interactome were Ras superfamily proteins (10 proteins), mainly members of the Rab family. Phosphodiesterases (four proteins) were the second largest group of the fish-parasite interactome. The detailed results of identification of proteins involved in potential host-parasite interactions are presented in Supplementary Files S1.19–20.

## 3. Discussion

This study is the first global proteomic analysis of the *A. simplex* (s.s.) L3 larval secretome. Previous proteomic investigations of *Anisakis* did not include profiling of the secretome proteins [13,31,32,33,34,35,36,37,38]. Therefore, knowledge on *A. simplex* ES proteins is very fragmented, although important aspects related to metabolism, pathogenicity, and host-parasite interactions are known to be associated with ES proteins. In this study, LC-MS/MS and bioinformatics analyses were applied to provide insights into these issues.

Prior to identifying *Anisakis* ES proteins by mass spectrometry, their SDS-PAGE profile was analyzed. Electrophoretic analysis confirmed the distribution of protein bands over a wide range of molecular weights, characteristic of the ES proteins of *A. simplex* L3 (see Supplementary Figure S1). Subsequently, LC-MS/MS analysis allows identification of 158 proteins in the *Anisakis* secretome, which is currently the largest proteomic dataset of *A. simplex* ES proteins. The number of ES proteins identified in this study corresponds to approximately 0.8% of the genes encoding *A. simplex* proteins. Comparing the number of identified ES proteins of *A. simplex* to the secretomes of other closely related pathogenic nematodes with similar genome size, such as *A. suum* and *T. canis* (see Figure 1), reveals that there is a relatively high number of ES proteins in *Anisakis*. By contrast, the lower number of identified *Anisakis* ES proteins compared to the *A. caninum* secretome is presumably due to the much larger genome size of *A. caninum*. Furthermore, only about one-quarter of the identified *Anisakis* ES proteins showed high similarity to the proteins of the *Toxocara* or *Ascaris* secretomes. This relatively low similarity is probably due to differences in hosts and life cycles of *Anisakis* nematodes and those of *Toxocara* and *Ascaris*.

The majority of detected *Anisakis* ES proteins were assigned to an unconventional secretory pathway (approximately 49% of proteins). This prediction is consistent with the secretory pathway analysis of ES proteins of other nematodes, such as *Dirofilaria immitis* [39] or *Strongyloides venezuelensis* [40]. Most secretome proteins of these nematodes were also classified into the unconventional secretory pathway. Furthermore, 15% of *Anisakis* proteins were classified as potentially EV-associated proteins. Prediction of these proteins was based on similarity to EV-associated proteins secreted by other nematodes. This is a particularly important identification because, among other considerations, the EV released by parasites play an important role in delivering molecules that can modulate the host immune response or the transfer of pathogenicity-related factors [28]. In this study, six EV-associated proteins were found which were previously identified by Boysen et al. [27] (see Section 2.4). There are currently no other published studies on the identification of *Anisakis* EV-associated proteins, and because of their important functions, this topic requires further exploration.

The secretome proteins detected in *Anisakis* were characterized by high diversity, as evidenced by their classification into 143 protein families. Among the most frequently identified protein families in the *Anisakis* secretome, attention should be paid to the annexin superfamily. Annexins have multiple functions, such as in cellular anti-inflammation, signal transmission, anticoagulation, ion channel regulation, membrane repair, and membrane transport, and likely participate in cell proliferation, differentiation, and apoptosis [41,42]. Furthermore, parasite annexins are considered potential drug and vaccine targets [43]. The thioredoxin-like superfamily is another of the protein families most frequently detected in the *Anisakis* secretome that is also important. Thioredoxins, inter alia, regulate thiol-based redox control and prevent the aggregation of cytosolic proteins in the cell [44,45]. The extracellular activities of thioredoxins include anti-inflammatory and antiapoptotic activities and, thus, cytoprotective effects [44,46].

In general, the identified ES proteins of *A. simplex* (s.s.) have multiple functions, as demonstrated by the GO analysis. On average, nine GO terms were detected for all 142 proteins that were assigned GO annotations. A large variety of GO terms among nematode secretome proteins is quite typical [47]. Many of the detected secretome proteins, such as thioredoxins [44], annexins [48], and HSPs [49], are moonlighting proteins that form a subset of multifunctional proteins in which one polypeptide chain exhibits more than one physiologically relevant biochemical or biophysical function [50]. 

GO annotation enrichment analysis provided interesting data. Of the many enriched annotations, the most abundant and most enriched GO terms were, in general, related to the glycolytic process, larval development, antioxidants, and cuticle. These annotations cover functions that are important to parasite metabolism, lifestyle, and survival, and they are also found to be enriched in the annotated secretomes of other nematodes [40,47]. Among the enriched GO terms mentioned above, those related to the cuticle may seem to be unassociated with the secretome. However, it should be noted that ES proteins are also released from the surface of the cuticle, in addition to specialized excretory-secretory organs and parasite intestine [24]. Proteins produced and presented at the parasite-host interface during invasion play a critical role in the induction and development of immune responses [24]. Furthermore, secretome proteins could also play essential roles in ensuring cuticle integrity [51].

Possible enzymes were detected among secretome proteins, which is in line with previous studies that confirmed the enzymatic properties of the *A. simplex* secretome [52]. Furthermore, Kim et al. [53] found that protease related genes are highly expressed in the transcriptome of *A. simplex* L3 larvae. In the present study, proteases were highly represented in the secretome (17% of ES *Anisakis* proteins). These enzymes are known to be especially important in the pathogenesis of anisakiasis and other parasitoses [54,55]. Proteases play an important part in host-parasite interactions, such as invasion of the host, migration through host tissues, protection of the parasite against the host immune system, and activation of the inflammatory response [56,57]. Proteases also participate in important biological processes in parasitic nematodes, as they are directly involved in their growth and survival, embryonic development, digestion of protein for nutrients, molting, and numerous metabolic processes [58,59]. Another important enzyme group detected in the secretome is antioxidant enzymes, such as thioredoxin-dependent peroxiredoxin and superoxide dismutase. These enzymes are found to be enriched in the secretome, and they function to protect against the toxic contents released by immune effector cells as a first-line host defense mechanism [22]. Acetylcholinesterase (AChE) was also detected as an enriched enzyme in the secretome. The enrichment of AChE in the *Anisakis* secretome is in agreement with previous investigations [60]. AChE secretion by *Anisakis* larvae is presumed to be an adaptive mechanism, and its secretion increases in response to a direct and/or indirect effect of neurotoxic compounds released by the host [60]. Furthermore, AChE has recently received attention as a potential anthelmintic drug and vaccine target in nematodes [61].

Among the KEGG pathways, an important group that was enriched comprises the following carbohydrate metabolism pathways: glyoxylate and dicarboxylate metabolism, propanoate metabolism, glycolysis/gluconeogenesis, and pentose phosphate pathway. Indeed, carbohydrate metabolism is an essential energy source for *Anisakis* larvae [62]. Carbohydrates play important roles in many basic processes, including development, morphogenesis, immunity, and host-pathogen interactions [63]. Two sugars, trehalose and glycogen, were detected in *A. simplex* L3 larvae [64]. Łopieńska-Biernat et al. found [65] that trehalose plays a key role in providing energy during thermotolerance and starvation processes. It is also worth mentioning that the secretome was enriched in numerous members of the longevity-regulating pathway, which is associated with the regulation of numerous processes, such as oxidative stress, autophagy, glycogen accumulation, and fat accumulation. In addition, the secretome was found to be enriched in proteins belonging to the valine, leucine, and isoleucine degradation KEGG pathway. This KEGG pathway is important since valine, leucine, and isoleucine are likely to be essential amino acids in *Anisakis*, as is the case in *C. elegans* [66].

Approximately 21% of ES proteins were predicted to be essential for life. These are proteins that are critical to the survival of the cell or organism under certain conditions [67]. Among the top matches of secretome essential proteins such proteins were found as putative actin (A0A0M3J0M4), calmodulin (A0A0M3KFJ2), elongation factor 2 (A0A0M3K613), and HSPs (A0A0M3K5H6, A0A0M3K9V2, A0A0M3K4G1). Actin is a family of globular multi-functional proteins that form microfilaments [68]. These proteins participates in many important cellular processes, including muscle contraction, cell motility, cell division and cytokinesis, vesicle and organelle movement, cell signaling, and the establishment and maintenance of cell junctions and cell shape [68]. Calmodulin is a highly conserved protein ubiquitously and abundantly expressed in eukaryotic cells [69]. The functions of calmodulin include Ca^2+^ binding and alteration of calcium signal transduction pathway to control a variety of biological processes, such as cytoskeletal assembly/reorganization, activation of phosphorylase kinase, abiotic stress responses, neurotransmission, smooth muscle contraction, metabolism, and cell motility [70]. Elongation factor 2 catalyzes the guanosine triphosphate-dependent ribosomal translocation step during translation elongation. HSPs are important molecular chaperones for maintaining cellular functions to prevent proteins from misfolding and aggregating in crowded surroundings. HSP expression levels increase when the organism is exposed to stress conditions, such as heat shock, alkaline treatment, and some chemical reagents, in order to help pathogens survive unfavorable conditions in the host [71,72,73]. Furthermore, essential for life proteins may be promising therapeutic targets for drugs and vaccines [74]. Such proteins include phosphoglycerate mutase (A0A0M3KAY8), which in this study was predicted to be an essential protein and shows homology with cofactor-independent phosphoglycerate mutase (iPGM) of *C. elegans*. This enzyme is involved in glycolytic and gluconeogenic pathways, and inhibition of iPGM activity has been shown to have a lethal effect on *C. elegans* [75]. Therefore, iPGM is considered a potential drug target or vaccine candidate in several nematodes, such as *Wuchereria bancrofti* [76], *B. malayi* [77], and *Leishmania donovani* [78].

Nine potential pathogenicity-related proteins in the *A. simplex* (s.s.) secretome were identified using database searching. The majority were HSPs which are the first line of attack and help in fortifying pathogen virulence [79,80]. The HSPs of *Anisakis* are poorly characterized; to date, only the expression patterns of HSP90 and HSP70 in *Anisakis* have been analyzed [81,82], whereas the contribution of *Anisakis* HSPs to pathogenicity has not been investigated. By contrast, HSP70 has a better-known role in the pathogenicity of *Toxoplasma gondii*, which is based on the modulation of nitric oxide production by macrophages [83]. HSP70 of *T. gondii* was found to be homologous with two top-matched potential pathogenicity-related proteins in the *A. simplex* secretome. Another two predicted pathogenicity-related proteins of *A. simplex* (s.s.) were homologous with GroEL of *Bartonella*, which is a chaperonin and exhibits pathogenicity via apoptosis inhibition and mitogenic stimulation of host cells [84]. The next two potential pathogenicity-related proteins in the *Anisakis* secretome were found to be homologous with thiol-specific antioxidant protein 1 of *Cryptococcus*, which is essential for resistance to oxidative, nitrosative, and temperature stress [85]. A homolog of hypothetical protein CNAG_05449 of *Cryptococcus* was also detected in the *Anisakis* secretome. This protein is in the metallothionein family, members of which play a crucial role in the pathogenicity and resistance of *Cryptococcus* against the host immune response, since they are directly involved in the detoxification of high concentrations of copper produced by macrophages fighting the infection [86]. Another protein detected in the *Anisakis* secretome is a homolog of the glycine cleavage system H protein of *Francisella tularensis*, which contributes to the intracellular replication of the pathogen in serine-limiting environments [87]. Another predicted pathogenicity-related protein of the *A. simplex* (s.s.) secretome is homologous with glucose-6-phosphate isomerase, which is required for the extracellular polysaccharide biosynthesis of *Haemophilus influenzae* [88].

Of the seven *A. simplex* ES allergens listed by WHO/IUIS [89], only Ani s 4 was detected in the present study. Ani s 4 is significant because of its heat- and pepsin-resistant properties and its ability to cause anaphylaxis [13,90,91]. Other *Anisakis* allergens were presumably not expressed in the in vitro culture conditions, or their concentration was below the limit of detection of LC-MS/MS. In addition to known allergens, potential allergens were also identified in this study. In order to increase the specificity of this analysis, hits detected using the FARRP database were confirmed by the AllerCatPro server [92], combining various bioinformatics approaches. The majority of the potential allergens detected in the *Anisakis* secretome are homologs of nematode and arthropod allergens, which is in line with the cross-reactions between *Anisakis* antigens and these organisms described by other authors [93,94,95]. Of the 18 potential allergens identified in this study, the following 6 were detected in our previous investigations of extracts from *A. simplex* L3 larvae: A0A158PP35, A0A0M3K5H6, A0A0M3KA05, A0A0M3JU57, A0A0M3K9V2, and A0A0M3K8L6 [13,33]. The first five are also proteins with potential thermostability [13]. Furthermore, many of the possible allergens detected in the *Anisakis* secretome show similarity to the potential allergens identified by other authors in the whole proteome and transcriptome of *Anisakis* larvae [32,96,97]. Fæste et al. [37] found in the *A. simplex* larvae the following proteins, including potential allergens which were similar to putative allergens identified in the present study: haemoglobin (P26914), troponin-like protein (Q9U3U5), HSP 70 (A8Q5Z6), triosephosphate isomerase (P91919), fructose-bisphosphate aldolase 1 (A8P3E5), and calmodulin (O16305). In particular, many sequences similar to the potential allergens of *A. simplex* detected in this study can be found at the transcriptome level in the ANISAKIS DB database (http://anisakis.mncn.csic.es/public/, accessed on 17 January 2021) [97]. Furthermore, a comparison of the secretome proteins identified in this study with the immunoreactive proteins of *A. simplex* esophageal gland cells provides interesting data [38]. Of the 13 immunoreactive proteins detected in esophageal gland cells, we also detected the following 4 in the secretome: uncharacterized protein (A0A0M3K6E2), uncharacterized protein (A0A0M3JQQ1), metalloendopeptidase (A0A0M3K299), and SCP domain-containing protein (A0A0M3K1U4). Thus, these ES proteins may also have allergic and/or diagnostic potential.

Interactome analysis was performed to identify and characterize proteins involved in interactions between *A. simplex* (s.s.) ES proteins as well as proteins involved in host-pathogen interactions. As was expected, such proteins primarily fall into the following groups: proteins of KEGG pathways, essential proteins, and proteases/protease inhibitors, followed by potential allergens and potential pathogenicity-related proteins. These groups of *Anisakis* proteins are characterized in detail above, and such profile composition is in accordance with the main functions of helminth ES proteins, i.e., penetration, colonization, survival in host tissues, incorporation of host metabolites, and modulation of the host immune response [98,99]. 

Among the human proteins involved in the interaction with *Anisakis* ES proteins, laminins were found to be the most highly represented. Laminins, which are the major component of the basal lamina, are enzymatically degraded by parasites such us *Anisakis pegreffii* during invasion [100], which facilitates internal migration of parasites [101]. Methyltransferase proteins were also highly abundant human proteins which were predicted in the host-parasite interactome. These proteins are known to contribute in deregulation of host expression profile which lead to host cell transformation, or escape of Apicomplexa parasites from the host immune system [102]. It is worth noting that among all human-parasite protein interactions, human polyubiquitin-C is the main target identified as interacting with *Anisakis* ES proteins. Indeed, ubiquitin is known to modulate host-pathogen interactions, with a particular focus on host innate immune defenses and pathogen immune evasion [103]. Among *Anisakis* secretome proteins, transaldolase (A0A0M3KAE3) was found to interact with the largest number of human proteins. This protein is an enzyme of pentose phosphate pathway, and a potential allergen. Another *A. simplex* (s.s.) protein which was predicted to interact with a large number of human proteins is proteasome subunit alpha type (A0A0M3JT99) which is characterized by its proteolytic activity. Another important *Anisakis* protein of predicted human-parasite interactome is heat shock 70 kDa protein cognate 1 (A0A0M3K9V2). This protein was predicted to be a potential protease, a potential allergen, a protein essential for life, and a possible pathogenicity-related protein. It is known that heat shock 70 kDa cognate plays an important role in the interactions of many parasites with the host organism, since it is highly immunogenic and a target of B and T cells [104,105]. 

The number of proteins involved in the human-parasite interaction network is about four times the number of proteins in the fish-parasite interactome. This result could be mainly due to the fact that the available database of the non-human host-pathogen interaction is much more limited than for human-pathogen interactions. Among the fish proteins involved in the interaction with *Anisakis* secretome proteins, Rab-family proteins were found to be the most abundant. These proteins regulate virtually all membrane trafficking events in eukaryotic cells [106,107]. Other abundant proteins in fish-parasite interactome were phosphodiesterases (PDEs). PDEs are metallohydrolases that control the concentration of second messengers cyclic adenosine monophosphate and cyclic guanosine monophosphate. Among *Anisakis* secretome proteins, Rab GDP dissociation inhibitor (GDI) (A0A0M3JZR1) was found to interact with the largest number of fish proteins. This protein is involved in regulation of GDP-GTP exchange between Rab-family proteins. Rab GDI is an immunoreactive protein of *Trichinella britovi* [108], but its role in host-parasite interactions is poorly known. Similarly, calmodulin (A0A0M3KFJ2) of *Anisakis* secretome was predicted to interact with fish proteins. Furthermore, calmodulin it is known immunogenic protein of *Fasciola hepatica* secretome [109].

## 4. Materials and Methods

### 4.1. A. simplex (s.s.) L3 Larvae Collection and Identification

*Anisakis* spp. L3 larvae were collected from naturally infected Atlantic mackerel (*Scomber scombrus*) originated from subarea VII of FAO fishing area 27. As described in our previous publication [110], the larvae were purified by washing with sterile phosphate-buffered saline (PBS, 0.01 M, pH 7.4; Sigma, St. Louis, MO, USA) and microscopically evaluated for integrity and viability. Ten randomly selected larvae were used for identification of *Anisakis* species by PCR-restriction fragment length polymorphism (PCR-RFLP) [111,112,113,114] (see Supplementary Figure S2).

### 4.2. ES Proteins Preparation

ES proteins were prepared by incubating 100 viable L3 *A. simplex* (s.s.) larvae in 5 mL of sterile PBS. After 24 h of incubation at 36 °C, medium containing ES proteins was collected and clarified by centrifugation at 20,000× *g* for 15 min at 4 °C. Supernatants were concentrated by ultrafiltration at 4 °C (3 kDa cutoff membrane; Thermo Fisher Scientific, Rockford, IL, USA). Subsequently, protein concentration was measured using a spectrophotometer (NanoPhotometer P330, Implen, München, Germany) and secretome samples were stored at −80 °C for further experiments. According to this procedure, three independent biological replicates of ES proteins were prepared.

### 4.3. SDS-PAGE

ES proteins of *A. simplex* (s.s.) were subjected to 4–20% SDS-PAGE (BioRad, Hercules, CA, USA) under reducing conditions [115]. Gel was fluorescently stained with SYPRO Ruby protein gel stain (Invitrogen, Eugene, OR, USA) and the molecular weights of the electrophoresis bands were calculated using Bio-1D software (ver. 15.07; Vilber Lourmat, Marne-la-Vallée, France) (see Supplementary Figure S1).

### 4.4. Sample Processing and LC-MS/MS Analysis

Three batches of *A. simplex* (s.s.) ES proteins were subjected to in-solution digestion and LC-MS/MS analysis, as described in our previous publication [13]. Briefly, secretome samples were analyzed using a Q Exactive mass spectrometer (Thermo Electron Corp., San Jose, CA, USA) coupled with nano-high-performance liquid chromatography (nano-HPLC) RP-18 column (internal diameter 75 µm; Waters, Milford, MA, USA). Proteins were identified with Mascot search engine server (ver. 2.5; Matrix Science, London, UK; http://www.matrixscience.com/server.html, accessed on 25 October 2019) using the *A. simplex* proteome (proteome ID: UP000036680; 20,789 sequences) obtained from the Universal Protein Resource (UniProt, http://www.uniprot.org/, accessed on 25 October 2019) [116]. The following Mascot search parameters were applied: trypsin digestion allowing one missed cleavage, parent ions was set to 5 parts per million (ppm), and fragment ions was set to 0.01 dalton (Da). The ion type was set as monoisotopic, and protein mass was set as unrestricted. Beta-methylthiolation of cysteine was used as a fixed modification, whereas oxidation of methionine was set as a variable modification. Peptides were accepted at False Discovery Rate (FDR) ≤ 0.98%, ion score ≥ 38, and significant threshold of *p* ≤ 0.00026. Only proteins detected with at least one unique peptide and proteins identified in all three biological replicates were accepted for further analysis. A detailed procedure of samples processing and LC-MS/MS analysis is presented in Supplementary File S2.

### 4.5. Bioinformatics Analysis

Theoretical pI and MW values of ES proteins were derived from the Mascot server. Gene ontology (GO) analysis, InterPro protein family classification, and enzyme identification were performed using OmicsBox with the Blast2GO algorithm (ver. 1.4.12; BioBam Bioinformatics SL, Valencia, Spain, https://www.biobam.com/omicsbox/) [117]. Analyses were run with the default settings and filtered using nematode taxonomy, as previously described [13]. Proteases and protease inhibitors were identified using a BLASTP search against the MEROPS database (release 11 September 2020; https://www.ebi.ac.uk/merops/, accessed on 8 April 2021) [118]. Proteins were assigned to the conventional or unconventional secretory pathway using OutCyte 1.0 server (http://www.outcyte.com/, accessed on 1 April 2021) [119]. Putative EV-associated proteins were identified using a BLASTP search against the known EV-associated proteins detected in the secretomes of *A. suum* [28], *B. malayi* [29], and *N. brasiliensis* [30]. Similarities between *A. simplex* ES proteins and previously identified secretome proteins of the parasites *S. lupi* L3 larvae [26], adult *A. caninum* [25], *A. suum* L3 larvae [24], and *T. canis* larvae [23] were evaluated using BLASTP. The same bioinformatics tool was used to identify proteins essential for life using reference eukaryote proteins from the DEG database (release 1 September 2020; http://origin.tubic.org/deg/public/index.php/index, accessed on 22 April 2021) [67].

Enriched GO annotations and enzymes of identified secretome proteins were compared with those of the whole proteome of *A. simplex* (proteome ID: UP000036680) by two-tailed Fisher’s exact test using OmicsBox software. These tests were performed using a *p*-value cutoff of 0.05 to indicate significance. Proteins involved in KEGG pathways were detected using the KOBAS 3.0 server (http://kobas.cbi.pku.edu.cn/, accessed on 23 April 2021) [120] based on the *C. elegans* proteome as a reference. Enriched KEGG pathways were identified by Fisher’s exact test using KOBAS 3.0 with the *C. elegans* proteome as a reference and the whole *A. simplex* proteome as background. In this case, a *p*-value less than 0.05 with Benjamini and Hochberg correction was considered to indicate significance.

Potential pathogenicity-related proteins were predicted using BLASTP against the following databases: ProtVirDB (http://bioinfo.icgeb.res.in/protvirdb/home.html, accessed on 22 April 2021) [121], Victors (http://www.phidias.us/victors/, accessed on 23 April 2021) [122], and VFDB (http://www.mgc.ac.cn/VFs/main.htm, accessed on 23 April 2021) [123]. Proteins were evaluated for potential allergenicity by BLASTP searching against The Food Allergy Research and Resource Program (FARRP) AllergenOnline.org database (ver. 21; http://www.allergenonline.com/, accessed on 30 March 2021). Possible allergens detected using the FARRP database were confirmed by the AllerCatPro server (ver. 1.8; https://allercatpro.bii.a-star.edu.sg/, accessed on 10 April 2021) [92]. Unknown 3D structures of pathogenicity-related/potential pathogenicity-related proteins and allergens/potential allergens were predicted by homology modeling using the Phyre2 server (http://www.sbg.bio.ic.ac.uk/phyre2/, accessed on 9 June 2021) in intensive mode (multi-template + ab initio) [124]. The model structures were further improved using 3Drefine (http://sysbio.rnet.missouri.edu/3Drefine/, accessed on 13 June 2021) [125]. Visualization of the 3D structures of proteins was performed using the PyMOL Molecular Graphics System (ver. 2.0; Schrödinger, LLC, New York, NY, USA).

Interactions between *A. simplex* proteins were predicted with high confidence (score of at least 0.7) using the STRING server (ver. 11.0; https://string-db.org/, accessed on 20 June 2021) [126] based on known interactions of *C. elegans* orthologs. Host-parasite protein interactions were predicted using the HPIDB 3.0 server (https://hpidb.igbb.msstate.edu/hpi30_index.html, accessed on 12 December 2021) [127,128], which was run with the default setting using *A. simplex* (s.s.) ES proteins searched against human (*Homo sapiens*, UniProt proteome ID: UP000005640) and Atlantic herring (*Clupea harengus*, UniProt proteome ID: UP000515152) proteomes. Interactome networks were visualized and analyzed using Cytoscape software ver. 3.9.0 [129].

All BLASTP analyses in this study were performed using the OmicsBox software. Only hits with a BLAST e-value ≤ 1.0 × 10^−5^ and similarity ≥70% were considered. The databases, software tools, and servers used for bioinformatics analyses in this study are listed in Supplementary Table S1.

## 5. Conclusions

Proteomic analysis was performed for the first broad-scale identification and characterization of ES proteins of *A. simplex* (s.s.) L3 larvae. A total of 158 proteins, belonging to 143 different proteins families, were identified in *Anisakis* secretome using mass spectrometry technique. Comparison of *Anisakis* secretome proteins with ES proteins of closely related nematodes revealed that the *A. simplex* secretome contains a relatively high number of proteins with a low level of overall similarity to ES proteins of related parasites. Prediction of secretory pathways allowed the classification of the majority of proteins (approximately 49% of ES proteins) to the unconventional route. In addition, six *Anisakis* proteins previously known to be associated with EVs were detected and 24 new possibly EV-associated proteins were predicted. GO annotations, KEGG pathways, and enzymes were assigned to ES proteins and enrichment analysis of these terms was performed by comparison with whole *A. simplex* proteome. The most enriched GO annotations were terms related to the glycolytic process, larval development, antioxidants, and cuticle, while among the KEGG pathways the main enriched group was associated with carbohydrate metabolism. Furthermore, proteases were found to be highly represented enzymes in the secretome (17% of ES proteins). Another finding was identification of essential proteins (approximately 21% of ES proteins) that are indispensable for the survival of an organism. Important findings were identification of pathogenicity-related proteins, allergens, and potential allergens. Nine potential pathogenicity-related proteins were predicted, which were mostly homologs of chaperones. Of all secretome proteins, one was identified as an allergen, which was Ani s 4, and 18 were putative allergens, most of which were homologs of nematode and arthropod allergens. Another finding was prediction of proteins possible involved in interactions between *A. simplex* ES proteins as well as proteins involved in interactions between hosts and parasite.

As summarized above detected ES proteins play an important role in many biological processes and provide a better understanding of *A. simplex* survival, development, and invasion strategy. In addition, the identified secretome proteins could be used as targets for new drugs, vaccines, and diagnostic assays. Nevertheless, it should be noted that functional analysis of ES protein was performed using a bioinformatics approach. Therefore, future in vitro and in vivo studies are needed to confirm our findings regarding the role of detected proteins.

## Figures and Tables

**Table 1:** The top 10 matches of putative proteins associated with extracellular vesicles identified in the secretome of *A. simplex* (s.s.) L3 larvae.

Secretome Proteins		Extracellular Vesicle-Associated Proteins			Blast Similarity (%)
UniProt Accession No.	Protein Name	UniProt Accession No.	Protein Name	Organism	
A0A0M3K144	Proteasome subunit alpha type	F1L7Z7	Proteasome subunit alpha type	*Ascaris suum*	98.79
A0A0M3JSH7	Proteasome subunit alpha type-3	F1LBE7	Proteasome subunit alpha type-3	*Ascaris suum*	94.59
A0A0M3JVA5	Triosephosphate isomerase	A0A0J9YA50	Triosephosphate isomerase	*Brugia malayi*	93.50
A0A0M3K0Q9	Chaperonin homolog Hsp-60, mitochondrial	A0A0N4YLK1	Chaperonin homolog Hsp-60, mitochondrial	*Nippostrongylus brasiliensis*	92.76
A0A0M3KAY8	Phosphoglycerate mutase	Q4VWF8	2,3-bisphosphoglycerate-independent phosphoglycerate mutase	*Brugia malayi*	92.33
A0A0M3JT99	Proteasome subunit alpha type	A0A0N4XV21	Proteasome subunit alpha type	*Nippostrongylus brasiliensis*	92.02
A0A0M3J0T6	14-3-3-like protein 2	A0A0N4XVA6	14-3-3-like protein 2	*Nippostrongylus brasiliensis*	90.94
A0A0M3K444	Fructose-bisphosphate aldolase	A0A0H5S7G0	Fructose-bisphosphate aldolase	*Brugia malayi*	90.93
A0A0M3K9Z9	Peroxiredoxin 1	A0A0N4XCN5	Peroxiredoxin 1	*Nippostrongylus brasiliensis*	90.72
A0A0M3K4G1	Uncharacterized protein	A0A0N4YLK1	Chaperonin homolog Hsp-60, mitochondrial	*Nippostrongylus brasiliensis*	90.71

**Table 2:** The top 10 matches of proteases/protease inhibitors detected in the secretome of *A. simplex* (s.s.) L3 larvae.

Secretome Protein		Protease/Protease Inhibitor				BLAST Similarity (%)
UniProt Accession No.	Protein Name	MEROPS Accession No.	MEROPS Classification	Activity	Organism	
A0A0M3K144	Proteasome subunit alpha type	MER1107563	Subfamily T1A non-peptidase homologues (T01.UNA)	Threonine protease	*Anisakis simplex*	100.00
A0A0M3JT99	Proteasome subunit alpha type	MER1107399	Subfamily T1A unassigned peptidases (T01.UPA)	Threonine protease	*Anisakis simplex*	100.00
A0A0M3JSH7	Proteasome subunit alpha type-3	MER1107379	Subfamily T1A non-peptidase homologues (T01.UNA)	Threonine protease	*Anisakis simplex*	100.00
A0A0M3K9E5	M20_dimer domain-containing protein	MER1107182	Subfamily M20F non-peptidase homologues (M20.UNF)	Metalloprotease	*Anisakis simplex*	100.00
A0A0M3JV41	M20_dimer domain-containing protein	MER1107451	pes-9 g.p. (*Caenorhabditis elegans*) (M20.A14)	Metalloprotease	*Anisakis simplex*	100.00
A0A0M3K810	Dipeptidase C	MER1107162	Subfamily M24B non-peptidase homologues (M24.UNB)	Metalloprotease	*Anisakis simplex*	100.00
A0A0M3JXL8	Uncharacterized protein	MER1107491	F54F11.2 g.p. domain 2 (*Caenorhabditis elegans*) (M13.A20)	Metalloprotease	*Anisakis simplex*	100.00
A0A0M3KCB1	Uncharacterized protein	MER1107243	Family M1 non-peptidase homologues (M01.UNW)	Metalloprotease	*Anisakis simplex*	100.00
A0A0M3K9X8	Carboxylic ester hydrolase	MER1107193	Family S9 non-peptidase homologues (S09.UNW)	Serine protease	*Anisakis simplex*	100.00
A0A0M3JYK8	COesterase domain-containing protein	MER1107514	Family S9 non-peptidase homologues (S09.UNW)	Serine protease	*Anisakis simplex*	100.00

**Table 3:** The top 10 matches of essential proteins detected in the secretome of *A. simplex* (s.s.) L3 larvae.

Secretome Protein		Essential Protein			Blast Similarity (%)
UniProt Accession No.	Protein Name	DEG Accession No.	Protein Name	Organism	
A0A0M3J0M4	Putative actin	DEG20290735	Actin gamma 1	*Homo sapiens*	99.10
A0A0M3KFJ2	Calmodulin	DEG20070098	Calmodulin CG8472-PA, isoform A	*Drosophila melanogaster*	98.08
A0A0M3K916	Uncharacterized protein	DEG20051541	Alpha-actinin-4 (Non-muscle alpha-actinin 4) (F-actin cross-linking protein)	*Mus musculus*	91.16
A0A0M3K5H6	78 kDa glucose-regulated protein	DEG20040148	Immunoglobulin binding protein mRNA	*Danio rerio*	89.98
A0A0M3K613	Elongation factor 2	DEG20280147	Translation elongation factor 2	*Bombyx mori*	88.93
A0A0M3K9V2	Heat shock 70 kDa protein cognate 1	DEG20330753	Heat shock protein family A (Hsp70) member 8	*Homo sapiens*	86.16
A0A0M3K9Z9	Peroxiredoxin 1	DEG20201416	Peroxiredoxin 2	*Homo sapiens*	85.49
A0A0M3KAY8	Phosphoglycerate mutase (2,3-diphosphoglycerate-independent)	DEG20020003	F57B10.3a	*Caenorhabditis elegans*	84.52
A0A0M3K4G1	Uncharacterized protein	DEG20290492	Heat shock protein family D (Hsp60) member 1	*Homo sapiens*	84.44
A0A0M3K8S1	Methylmalonyl-CoA mutase	DEG20051827	Methylmalonyl-CoA mutase, mitochondrial precursor (MCM) (Methylmalonyl-CoA isomerase)	*Mus musculus*	83.97

**Table 4:** Results of identification of putative pathogenicity-related proteins in the secretome of *A. simplex* (s.s.) L3 larvae.

Secretome Protein		Pathogenicity-Related Protein				Blast Similarity (%)
UniProt Accession No.	Protein Name	Database	NCBI Accession No.	Protein Name	Organism	
A0A0M3K9V2	Heat shock 70 kDa protein cognate 1	ProtVirDB	AAC72001	Hsp70	*Toxoplasma gondii*	88.24
		VICTORS	BAB20284	Hsp70	*Toxoplasma gondii*	79.64
A0A0M3K5H6	78 kDa glucose-regulated protein	ProtVirDB	AAC72001	Hsp70	*Toxoplasma gondii*	86.06
		VICTORS	BAB20284	Hsp70	*Toxoplasma gondii*	80.36
		VFDB	NP_219906.1	Molecular chaperone DnaK	*Chlamydia trachomatis* D/UW-3/CX	70.23
A0A0M3K4G1	Uncharacterized protein	VICTORS	YP_989430	Chaperonin GroEL	*Bartonella bacilliformis* KC583	79.22
		VFDB	YP_001039283	Chaperonin GroEL	*Ruminiclostridium thermocellum* ATCC 27405	76.17
A0A0M3IZ99	Glucose-6-phosphate isomerase	VFDB	NP_439722.1	Glucose-6-phosphate isomerase	*Haemophilus influenzae* Rd KW20	76.46
A0A0M3K0Q9	Chaperonin homolog Hsp-60, mitochondrial	VICTORS	YP_989430	Chaperonin GroEL	*Bartonella bacilliformis* KC583	74.07
		VFDB	YP_003454101	Molecular chaperone GroEL	*Legionella longbeachae* NSW150	73.48
A0A0M3K9Z9	Peroxiredoxin 1	VICTORS	AAP68994	Thiol-specific antioxidant protein 1	*Cryptococcus neoformans* var. *grubii*	72.77
A0A0M3K6L1	Superoxide dismutase [Cu-Zn]	VICTORS	XP_012053609	Hypothetical protein CNAG_05449	*Cryptococcus neoformans* var. *grubii* H99	71.96
A0A0M3KAE7	Glycine cleavage system H protein	VICTORS	YP_169453	Glycine cleavage system H protein	*Francisella tularensis* subsp. *tularensis* SCHU S4	71.30
A0A0M3J2W3	Probable peroxiredoxin prdx-3	VICTORS	AAP68994	Thiol-specific antioxidant protein 1	*Cryptococcus neoformans* var. *grubii*	70.27

**Table 5:** Results of identification of potential allergens in the secretome of *A. simplex* (s.s.) L3 larvae.

Secretome Protein		FARRP Database Match				AllerCatPro Prediction
UniProt Accession No.	Protein Name	NCBI Accession No.	Protein Name	Organism	Blast Similarity (%)	
A0A0M3KA05	SXP/RAL-2 family protein 2 isoform 1	BAF75681	SXP/RAL-2 family protein 2 isoform 1	*Anisakis simplex*	100.00	Strong evidence
A0A0M3KIW7	Globin-like protein	ASL68918	Hemoglobin	*Anisakis simplex*	100.00	Strong evidence
A0A0M3JEL6	Globin-like protein	ASL68918	Hemoglobin	*Anisakis simplex*	100.00	Strong evidence
A0A0M3JU57	Troponin-like protein	CAB58171	Troponin-like protein	*Anisakis simplex*	99.38	Strong evidence
A0A158PP35	Paramyosin	Q9NJA9	Paramyosin (Ani s 2)	*Anisakis simplex*	92.72	Strong evidence
A0A0M3K5H6	78 kDa glucose-regulated protein	ABF18258	Heat shock cognate 70	*Aedes aegypti*	89.69	Strong evidence
A0A0M3K8L6	Uncharacterized protein	Q06811	Polyprotein ABA-1	*Ascaris suum*	85.67	Weak evidence
A0A0M3J5J0	Uncharacterized protein	P46436	Glutathione S-transferase 1	*Ascaris suum*	82.63	Strong evidence
A0A0M3IZ99	Glucose-6-phosphate isomerase	XP_026782721	LOW QUALITY PROTEIN: glucose-6-phosphate isomerase b	*Pangasianodon hypophthalmus*	82.27	Strong evidence
A0A0M3K9V2	Heat shock 70 kDa protein cognate 1	AOD75395	Heat shock-like protein	*Tyrophagus putrescentiae*	81.52	Strong evidence
A0A0M3JVA5	Triosephosphate isomerase	AEB54655	Triosephosphate isomerase	*Procambarus clarkii*	80.74	Weak evidence
A0A0M3JT42	Peptidyl-prolyl cis-trans isomerase (PPIase)	AAP35065	Der f Mal f 6 allergen	*Dermatophagoides farinae*	78.26	Weak evidence
A0A0M3K444	Fructose-bisphosphate aldolase	XP_026771637	Aldolase a, fructose-bisphosphate, b	*Pangasianodon hypophthalmus*	76.32	Weak evidence
A0A0M3KFJ2	Calmodulin	ACL36923	Troponin C	*Tyrophagus putrescentiae*	76.00	Weak evidence
A0A0M3JYW9	Fructose-bisphosphate aldolase	ACH70901	Aldolase a, fructose-bisphosphate 1	*Salmo salar*	74.93	Strong evidence
A0A0M3K3X7	Inorganic diphosphatase	QAT18643	Allergen Der p 32	*Dermatophagoides pteronyssinus*	74.51	Strong evidence
A0A0M3KAE3	Transaldolase	AHY02994	Transaldolase	*Fusarium proliferatum*	71.16	Weak evidence
A0A0M3JTF7	Peptidase A1 domain-containing protein	XP_001657556	Lysosomal aspartic protease	*Aedes aegypti*	70.82	Weak evidence

## Data Availability

The mass spectrometry proteomics data have been deposited in the Zenodo with the digital object identifier (DOI) 10.5281/zenodo.5866516 (https://doi.org/10.5281/zenodo.5866516) accessed on 17 January 2022.

## Supplementary Materials

The following supporting information can be downloaded at: https://www.mdpi.com/article/10.3390/pathogens11020246/s1, File S1: 1. List of proteins identified in all three biological replicates of *A. simplex* secretome; 2. Mascot results of proteins identification within individual biological replicates of *Anisakis* secretome; 3. Results of the comparison of the similarity of secretome proteins of *A. simplex* (s.s.) L3 larvae with *S. lupi* L3 larvae ES proteins; 4. Results of the comparison of the similarity of secretome proteins of *A. simplex* (s.s.) L3 larvae with adult *A. caninum* ES proteins; 5. Results of the comparison of the similarity of secretome proteins of *A. simplex* (s.s.) L3 larvae with *T. canis* larvae ES proteins; 6. Results of the comparison of the similarity of secretome proteins of *A. simplex* (s.s.) L3 larvae with adult *A. suum* ES proteins; 7. Results of the comparison of the similarity of secretome proteins of *A. simplex* (s.s.) L3 larvae with *A. suum* L3 larvae ES proteins; 8. Results of the identification of secretome proteins of *A. simplex* (s.s.) L3 larvae shared with ES proteins of the following nematodes: adult *A. caninum*, adult *A. suum*, *A. suum* L3 larvae, *T. canis* larvae, and *S. lupi* L3 larvae; 9. Results of protein family identification; 10. Results of prediction of secretome proteins associated with extracellular vesicles; 11. Results of identification of proteins secreted through an unconventional pathway; 12. Results of identification of conventionally secreted proteins; 13. Results of protein functional annotation; 14. Results of GO enrichment analysis; 15. Results of enzyme enrichment analysis; 16. Results of protease and protease inhibitor identification using MEROPS database; 17. Results of KEGG pathway enrichment analysis; 18. Results of identification of proteins essential for life; 19. Results of identification of proteins involved in potential human-parasite interactions; 20. Results of identification of proteins involved in potential fish-parasite interactions. File S2: Procedure of sample processing and LC-MS/MS analysis. Table S1: The databases, software tools, and servers used for bioinformatics analyses in the present study. Figure S1: Secretome protein analysis of *A. simplex* (s.s.) L3 larvae using SDS-PAGE stained with SYPRO Ruby. Molecular weight (MW) estimations are presented in kilodaltons (kDa). Figure S2: Restriction fragment length polymorphism patterns of the ITS region of the rDNA of *Anisakis* L3 larvae using the *Hinf*I (A) and *Hha*I (B) restriction enzymes.
