# Supplementary material for: Proteomic Profiling and In Silico Characterization of the Secretome of Anisakis simplex Sensu Stricto L3 Larvae
Source: Pathogens. 2022 Feb 14;11(2):246. doi: 10.3390/pathogens11020246 (PMC8879239; doi:10.3390/pathogens11020246)
Supplement: Supplementary file 1 [file pathogens-11-00246-s001.zip › Supplementary File S2.pdf]

Procedure of sample processing and LC-MS/MS analysis

Three batches of *A. simplex* (s.s.) ES proteins were subjected to the standard procedure of tryptic digestion. ES proteins were reduced with 0.5 M tris (2-carboxyethyl) phosphine (TCEP; Sigma, St. Louis, MO, USA) for 1 h at 60 °C, and the cysteine residues were subsequently alkylated with 200 mM methyl methanethiosulfonate (MMTS; Sigma, St. Louis, MO, USA) for 10 min at room temperature and finally cleaved overnight with 10 µL of 0.1 µg/µL trypsin (Promega, Madison, WI, USA) at 37 °C. After digestion, the resulting peptide mixtures were loaded in equal volumes of 20 µL to a reversed-phase RP-18 pre-column (Waters, Milford, MA, USA) using 0.1% formic acid (FA; Sigma, St. Louis, MO, USA) in water as a mobile phase and then transferred to nano-high-performance liquid chromatography (nano-HPLC) RP-18 column (internal diameter 75 µm; Waters, Milford, MA, USA) using linear acetonitrile (ACN; Sigma, St. Louis, MO, USA) gradient 0–35% over 160 min in the presence of 0.1% FA at a flow rate of 250 nL/min. The nano-HPLC column outlet was coupled directly to the ion source of the Q Exactive mass spectrometer (Thermo Electron Corp., San Jose, CA, USA) working in the regime of data-dependent MS to MS/MS switch with higher-energy collisional dissociation (HCD) type peptide fragmentation. A blank run ensuring the absence of cross-contamination from previous samples preceded each analysis.

Mass spectrometric data were preprocessed with Mascot Distiller software (ver. 2.6; Matrix Science, London, UK; http://www.matrixscience.com/distiller.html) and analyzed using the Mascot search engine server (ver. 2.5; Matrix Science, London, UK; http://www.matrixscience.com/server.html) against the *A. simplex* proteome (proteome ID: UP000036680; 20,789 sequences) obtained from the Universal Protein Resource (UniProt, http://www.uniprot.org/). To reduce mass errors, the peptide and fragment mass tolerance settings were established separately for individual LC-MS/MS runs after a measured mass recalibration, resulting in values of 5 parts per million (ppm) for the parent and 0.01 dalton (Da) for fragment ions in higher-energy collisional dissociation (HCD) MS/MS mode. Peptide sequences were searched using trypsin specificity allowing 1 missed cleavage; the ion type was set as monoisotopic, and protein mass was set as unrestricted. Beta-methylthiolation of cysteine was used as a fixed modification, whereas oxidation of methionine was set as a variable modification. Peptides were accepted at False Discovery Rate (FDR) $\leq$ 0.98%, ion score $\geq$ 38, and significant threshold of $P \leq 0.00026$. Only proteins detected with at least one unique peptide and proteins identified in all 3 biological replicates were accepted for further analysis.
