# Supplementary material for: Proteomic Profiling and In Silico Characterization of the Secretome of Anisakis simplex Sensu Stricto L3 Larvae
Source: Pathogens. 2022 Feb 14;11(2):246. doi: 10.3390/pathogens11020246 (PMC8879239; doi:10.3390/pathogens11020246)
Supplement: Supplementary file 1 [file pathogens-11-00246-s001.zip › Supplementary Table S1.pdf]

Supplementary Table S1. The databases, software tools, and servers used for bioinformatics analyses in the present study.

Bioinformatics analysis of secretome proteins of *A. simplex*	Database	Bioinformatics tool
Estimation of theoretical isoelectric point	-[1]	Mascot server (ver. 2.5)
Estimation of theoretical molecular weight	-	Mascot server (ver. 2.5)
Gene ontology analysis	-	OmicsBox (ver. 1.4.12)
InterPro protein family classification	-	OmicsBox (ver. 1.4.12)
Enzyme identification	-	OmicsBox (ver. 1.4.12)
Identification of proteases and protease inhibitors	MEROPS database (release 12.3)	BLASTP search using OmicsBox (ver. 1.4.12)
Prediction of secretory pathway	-	OutCyte 1.0 server
Prediction of extracellular vesicles-associated proteins	Extracellular vesicles-associated proteins detected in the secretomes of *A. suum*, *B. malayi*, and *N. brasiliensis*	BLASTP search using OmicsBox (ver. 1.4.12)
Analysis of the similarity between *A. simplex* secretome proteins and secretome proteins of other nematodes	Secretome proteins of *S. lupi*, *A. caninum*, *A. suum*, and *T. canis*	BLASTP search using OmicsBox (ver. 1.4.12)
Prediction of proteins essential for life	Reference eukaryote proteins from the DEG database (release 09.01.2020)	BLASTP search using OmicsBox (ver. 1.4.12)
Gene Ontology enrichment analysis	*A. simplex* proteome (UniProt ID: UP000036680)	Two-tailed Fisher's exact test using OmicsBox (ver. 1.4.12)
Enzymes enrichment analysis	*A. simplex* proteome (UniProt ID: UP000036680)	Two-tailed Fisher's exact test using OmicsBox (ver. 1.4.12)
KEGG pathways identification	-	KOBAS 3.0 server
KEGG pathways enrichment analysis	*A. simplex* proteome (UniProt ID: UP000036680)	Fisher's exact test using KOBAS 3.0 server
Identification of potential pathogenicity-related proteins	ProtVirDB, Victors, VFDB	BLASTP search using OmicsBox (ver. 1.4.12)
Identification of potential allergens	Food Allergy Research and Resource Program (FARRP) AllergenOnline.org database (ver. 21)	BLASTP search using OmicsBox (ver. 1.4.12)
Confirmatory analysis of identified potential allergens	-	AllerCatPro server (ver. 1.8)
Prediction of 3D structures of proteins	-	Phyre2 server
Improvement of predicted 3D protein structures	-	3Drefine server
Visualization of predicted 3D protein structures	-	PyMOL Molecular Graphics System (ver. 2.0)
Prediction of interactions between *A. simplex* proteins	-	STRING server (ver. 11.0)
Prediction of host–parasite protein interactions	*Homo sapiens* proteome (UniProt ID: UP000005640); *Clupea harengus* proteome (UniProt ID: UP000515152)	HPIDB 3.0 server
Visualization and analysis of predicted interactomes	-	Cytoscape (ver. 3.9.0)

[1] Not applicable
