# Supplementary figures and images for: Proteomic Profiling and In Silico Characterization of the Secretome of Anisakis simplex Sensu Stricto L3 Larvae
Source: Pathogens. 2022 Feb 14;11(2):246. doi: 10.3390/pathogens11020246 (PMC8879239; doi:10.3390/pathogens11020246)

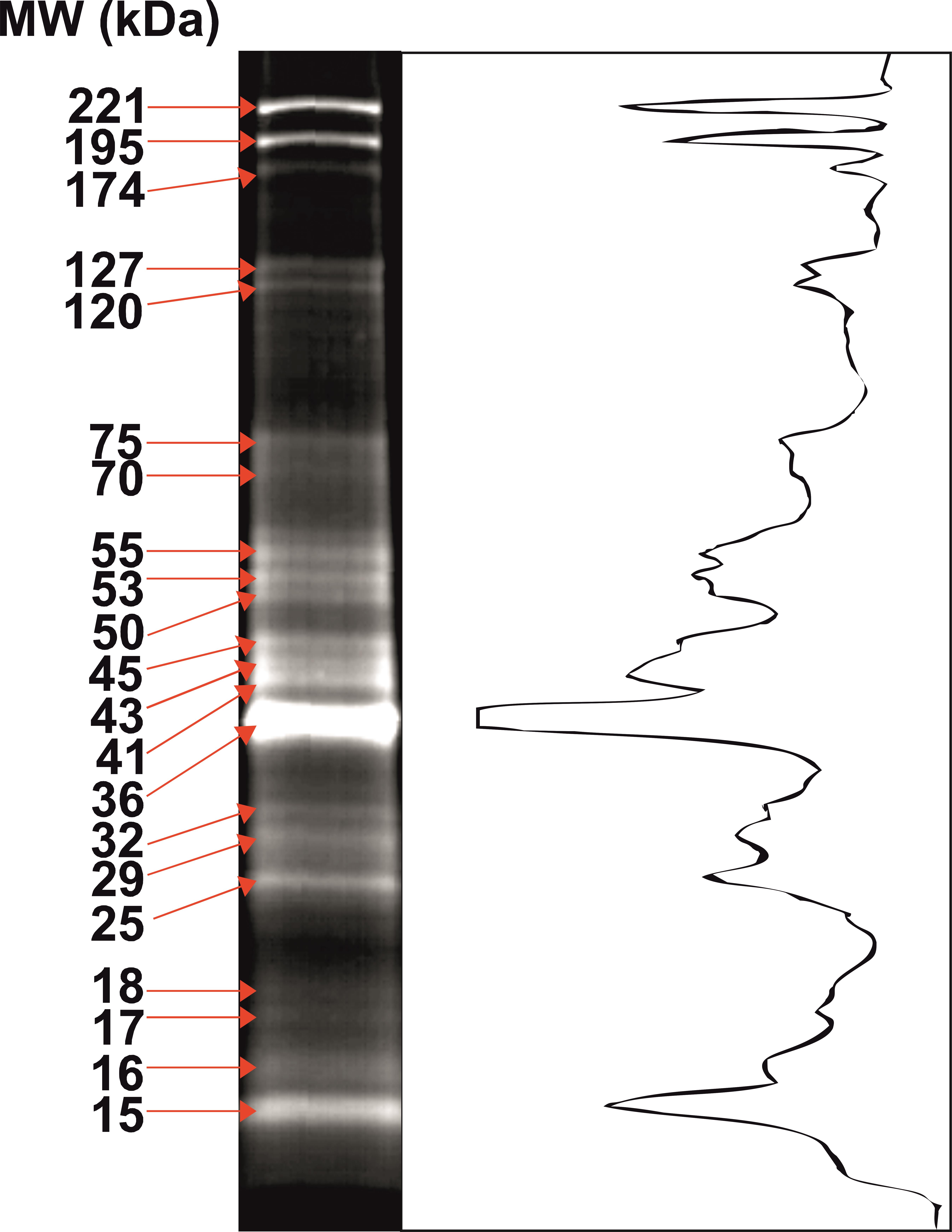

Supplement: Supplementary file 1 [file pathogens-11-00246-s001.zip › Supplementary Figure S1.jpg]
